# Thermoelectric materials taking advantage of spin entropy: lessons from chalcogenides and oxides

**DOI:** 10.1080/14686996.2021.1951593

**Published:** 2021-08-03

**Authors:** Sylvie Hébert, Ramzy Daou, Antoine Maignan, Subarna Das, Aritra Banerjee, Yannick Klein, Cédric Bourgès, Naohito Tsujii, Takao Mori

**Affiliations:** a Laboratoire de Cristallographie et Sciences des Matériaux (CRISMAT), Normandie Université, UMR6508 CNRS, ENSICAEN, UNICAEN, Caen, France; b Department of Physics, University of Calcutta, Kolkata, India; c IMPMC, Sorbonne Université, Paris, France; d International Center for Materials Nanoarchitectonics (WPI-MANA), National Institute for Materials Science, Tsukuba, Japan; e Graduate School of Pure and Applied Sciences, University of Tsukuba, Tsukuba, Japan

**Keywords:** Thermopower, chalcogenides, oxides, spins, entropy, magneto-resistance, 50 Energy Materials, 203 Magnetics / Spintronics / Superconductors, 206 Energy conversion / transport / storage / recovery, 210 Thermoelectronics / Thermal transport / insulators, chalcogenides, magneto-thermopower

## Abstract

The interplay between charges and spins may influence the dynamics of the carriers and determine their thermoelectric properties. In that respect, magneto-thermoelectric power MTEP, i.e. the measurements of the Seebeck coefficient *S* under the application of an external magnetic field, is a powerful technique to reveal the role of magnetic moments on *S*. This is illustrated by different transition metal chalcogenides: CuCrTiS_4_ and CuMnTiS_4_ magnetic thiospinels, which are compared with magnetic oxides, Curie-Weiss (CW) paramagnetic misfit cobaltites, ruthenates, either ferromagnetic perovskite or Pauli paramagnet quadruple perovskites, and CuGa_1-*x*_Mn*_x_*Te_2_ chalcopyrite telluride and Bi_1.99_Cr_0.01_Te_3_ in which diluted magnetism is induced by 3%-Mn and 1%-Cr substitution, respectively. In the case of a ferromagnet (below T_C_) and CW paramagnetic materials, the increase of magnetization at low T when a magnetic field is applied is accompanied by a decrease of the entropy of the carriers and hence $\left|S\right|$ decreases. This is consistent with the lack of MTEP in the Pauli paramagnetic quadruple perovskites. Also, no significant MTEP is observed in CuGa_1-*x*_Mn*_x_*Te_2_ and Bi_1.99_Cr_0.01_Te_3_, for which Kondo-type interaction between magnetic moments and carriers prevails. In contrast, spin glass CuCrTiS_4_ exhibits negative MTEP like in ferromagnetic ruthenates and paramagnetic misfit cobaltites. This investigation of some chalcogenides and oxides provides key ingredients to select magnetic materials for which *S* benefits from spin entropy.

## Graphical abstract

**Figure:**
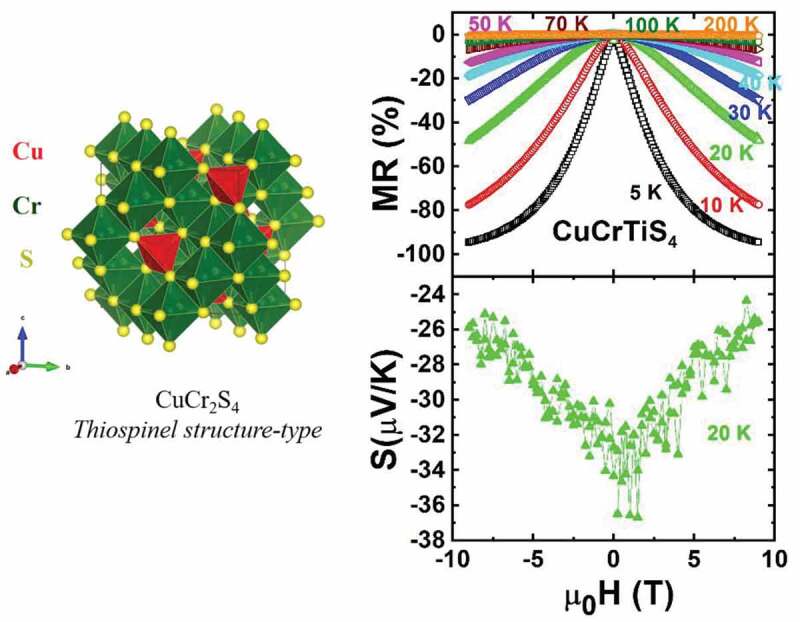

## 1. Introduction

**Figure 1.**
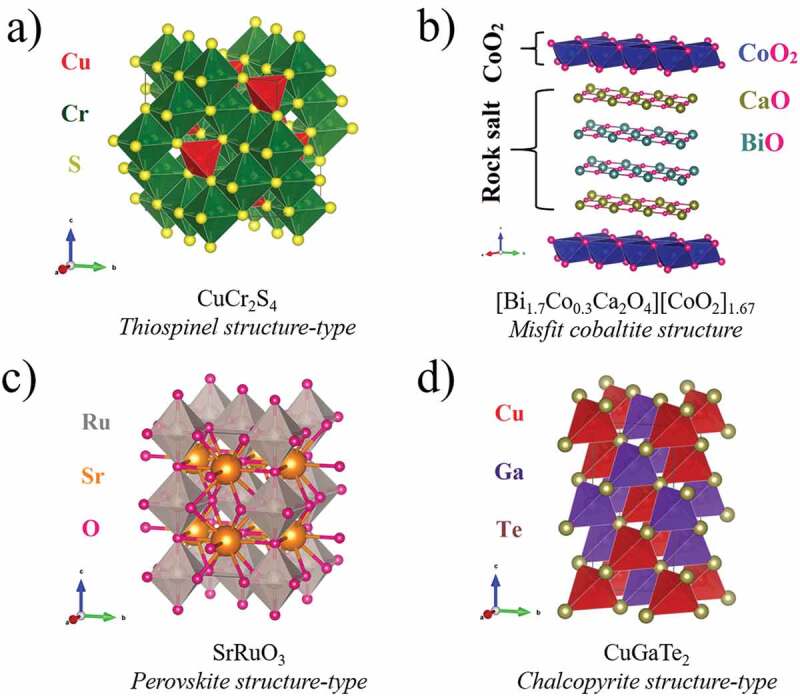
Schematic structural representation example of a) CuCr_2_S_4_ Thiospinel structure-type, b) Misfit Cobaltite structure, c) SrRuO_3_ Perovskite structure-type and d) CuGaTe_2_ Chalcopyrite structure-type

Thermoelectric (TE) materials have the potential to be one of the green solutions towards todays’ global energy crisis [1,2]. Over the years, to make TE devices a commercial success, many efforts have been made first to optimize material parameters like Seebeck coefficient (*S*) and electrical resistivity (ρ) through band engineering approaches, including band distortion [3], band convergence [4,5], and band nesting [6], to find a candidate with a large power factor PF as PF enters the TE Figure of merit *ZT* (= S^2^T/(ρκ) = (PF)T/κ), where к and T are the thermal conductivity and absolute temperature, respectively. Many chalcogenides having transition metal cations with partially filled d orbitals in them exhibit fascinating TE properties. In most cases, their interesting transport properties cannot be described by standard models. Strong correlations between carriers and interplay between charges and spins can influence the dynamics of the carriers and determine their TE properties [7–9]. Magnetic field-dependent thermoelectric power (MTEP) along with magnetoresistance (MR) studies provide a tool to unveil the possible mechanisms of unconventional transport properties of such magnetic systems and subsequently find new TE materials with suitable parameters. As the Seebeck coefficient depends on both transport coefficients and entropy [10], the application of a magnetic field can affect both terms. A significant MTEP usually signifies large orbital or spin degrees of freedom of the carriers, which through increasing entropy can result in large *S*, which is required for TE application [11]. At first, considering metal transition oxides, Wang et al., through MTEP measurement of Na*_x_*CoO_2_ samples with mixed valency of low spin Co^3+^ and Co^4+^, demonstrated that *S* is dominated by spin entropy in these layered oxides [8]. As for the latter system, the misfit cobalt oxides, another class of compounds containing CoO_2_ layers of the CdI_2_-type (Figure 1), exhibit a temperature dependence of *S*, which is not metal like. The low temperature slope of *S* in BiCaCoO misfit is strongly enhanced by the presence of paramagnetic spins as evidenced by its large negative MTEP below 20 K [12]. However, in oxides, the MTEP is not limited to CdI_2_-type structure in which the transition metal forms hexagonal layers but is also found in square lattices of 3D perovskites (Figure 1). A giant negative MTEP between −80% and −100% under 5 T in the 60–225 K temperature range was observed in the Nd_0.75_Na_0.25_MnO_3_ perovskite manganite and was accompanied by a large MR, which can be correlated with magnetic field-induced collapse of antiferromagnetism (AFM) [13]. It is noteworthy to mention that large MTEP and MR can also be observed without the presence of any magnetic cation in some topological insulators and phases having Dirac states [14–16]. Such effects arise from non-trivial band structure and are not in the scope of this study.

Apart from oxides, there exists other metal transition magnetic compounds containing chalcogen anions like S, Se and Te, which show excellent TE parameters with or without MTEP effect. For sulphides, considering the spinel structure first found in oxides, this is exemplified by the CuCrTiS_4_ thiospinel (Figure 1), characterized by a large TEP absolute value (*S*_300K_ = −180 µV/K), and for which MTEP was reported [17]. This large S absolute value is remarkable if one considers the low S values reported for the metallic behaviours of both end members CuCr_2_S_4_ and CuTi_2_S_4_ [17]. In some magnetic chalcopyrites also, where all metals are tetrahedrally coordinated to sulphur anions (Figure 1), magnetism was invoked to explain the large TEP. Compounds derived from AFM CuFeS_2_ chalcopyrite may exhibit a large power factor, by doping at the Cu site, or by creating S vacancies [18]. Indeed, a maximum power factor of 10^−3^ Wm^−1^K^−2^ was found for Cu_0.97_Zn_0.03_FeS_2_ where large TEP was explained through an enhancement of the effective mass m* due to the interaction between delocalized carriers and localized antiferromagnetic spins [19,20].

Here, we present an MTEP investigation of oxides and sulphides possessing different structures schematically described in Figure 1. The variety of their crystallographic structures, 2D or 3D, with magnetic cations forming hexagonal or cubic arrays and with very different metal coordinations, octahedral in misfit cobaltites and perovskite ruthenates, tetrahedral in chalcopyrites or both octahedral and tetrahedral coordinations in thiospinels, is at the origin of different electronic and magnetic states. In the following, the relationship between magnetism and thermopower is demonstrated in this diversity of thermoelectric compounds. Though fundamental, our comparative study will help to understand and correlate the interplay between spin and thermal transport in systems having different magnetic properties, which might be important for their further development towards better TE materials.

## 2. Experimental details

According to our previous studies [21–23], among magnetic chalcogenides, sulphides like CuCrTiS_4_ thiospinel and tellurides like CuGa_1-*x*_Mn*_x_*Te_2_ chalcopyrite (0 ≤ × ≤ 0.03) and Bi_1.99_Cr_0.01_Te_3_ were chosen. The synthesis of these materials was carried out using solid state reaction technique by heating the constituent elements under vacuum. A CuMn_0.5_Ti_1.5_S_4_ ceramic sample was also prepared replacing Cr by Mn/Ti metal and using the same synthesis method as in Ref [21]. As for oxides, three families were investigated with different magnetic properties: SrRuO_3_ perovskite, *D*Cu_3_Ru_4_O_12_ (*D*= Na, Ca, Ca_0.5_La_0.5_, La) quadruple perovskites, and misfit cobaltites like [Bi*A*_2_O_4_][CoO_2_]_b1/b2_ (*A* = Ca^2+^, Sr^2+^, Ba^2+^; b_1_/b_2_ = crystallographic misfit ratio) were taken for investigation. These oxides were prepared by standard ceramic method. The details of synthesis of the samples along with their in-depth structural characterization and experimental details about magnetic property measurements can be found elsewhere [21–26].

**Figure 2.**
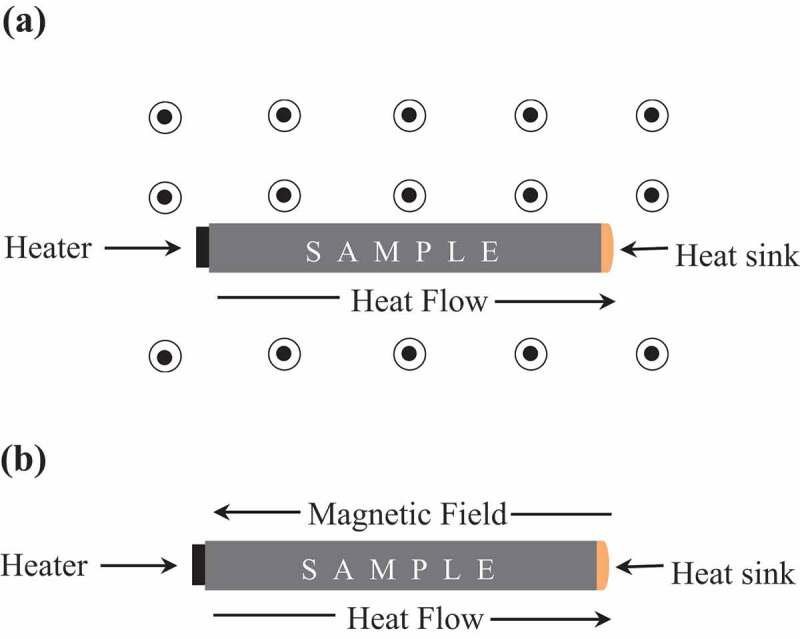
Schematic diagram of the MTEP set up in (a) homemade sample puck where direction of magnetic field is perpendicular to the direction of heat flow, as represented by the black circles (b) TTO option from Quantum Design where direction of heat flow and magnetic field is parallel to each other

Thermopower (TEP) measurements were performed using a homemade sample puck in a physical property measurement system (9 T-PPMS, Quantum Design, San Diego, USA) [27]. Measurements are performed using a four-point steady-state technique with separate measuring and power contacts. Bars of typical dimension of 2×2×10 mm^3^ were mounted using GE varnish on the heat sink of the cryostat, and two chromel-constantan thermocouples were attached to monitor the temperature gradient. The thermoelectric voltage was measured through the chromel wires. For MTEP measurements (Figure 2), magnetic field perpendicular to the temperature gradient was applied (Figure 2(a)). Measurements were done either in isothermal conditions by varying the magnetic field or as a function of temperature in an applied magnetic field. The thermoelectric properties of CuGa_1-*x*_Mn*_x_*Te_2_ and Bi_1.99_Cr_0.01_Te_3_ were measured using the TTO option from Quantum Design. For CuGa_1-*x*_Mn*_x_*Te_2_, magnetic fields were applied both parallel and perpendicular to the temperature gradient. As shown below, the two different configurations gave essentially the same results. Thus, for Bi_1.99_Cr_0.01_Te_3_, the magnetic field was applied parallel to the direction of heat gradient (Figure 2(b)).

MR was measured in the same instrument by a conventional four probe method by applying magnetic field perpendicular to the current flow. For CuGa_1-*x*_Mn*_x_*Te_2_ samples, MR measurements were performed by applying magnetic field both parallel and perpendicular to the current.

## 3. Results and discussions

### 3.1. Paramagnetic systems

**Figure 3.**
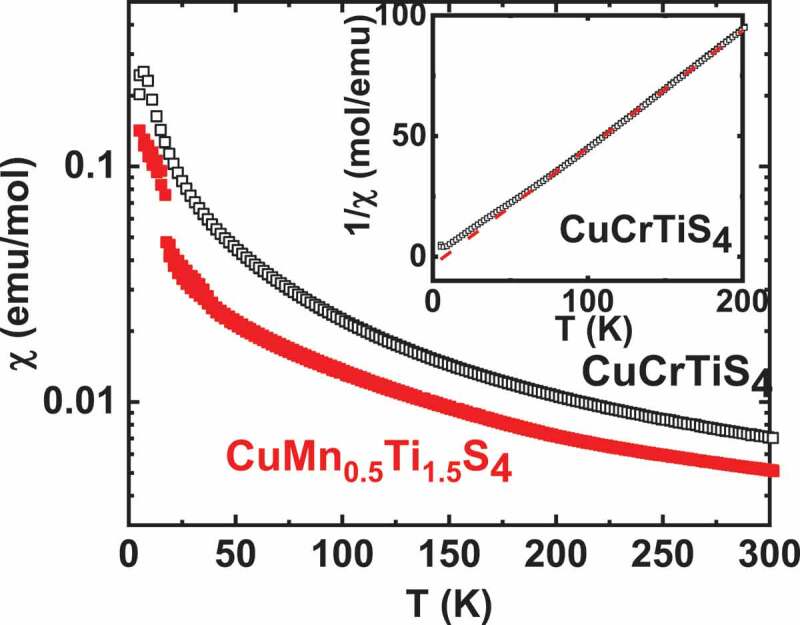
Temperature dependence of magnetic susceptibility (χ) of CuCrTiS_4_ and CuMn_0.5_Ti_1.5_S_4_ in a field of 10^−2^ T. Inset: χ^−1^ (T) of CuCrTiS_4_ where the dashed line corresponds to the Curie-Weiss fitting

**Figure 4.**
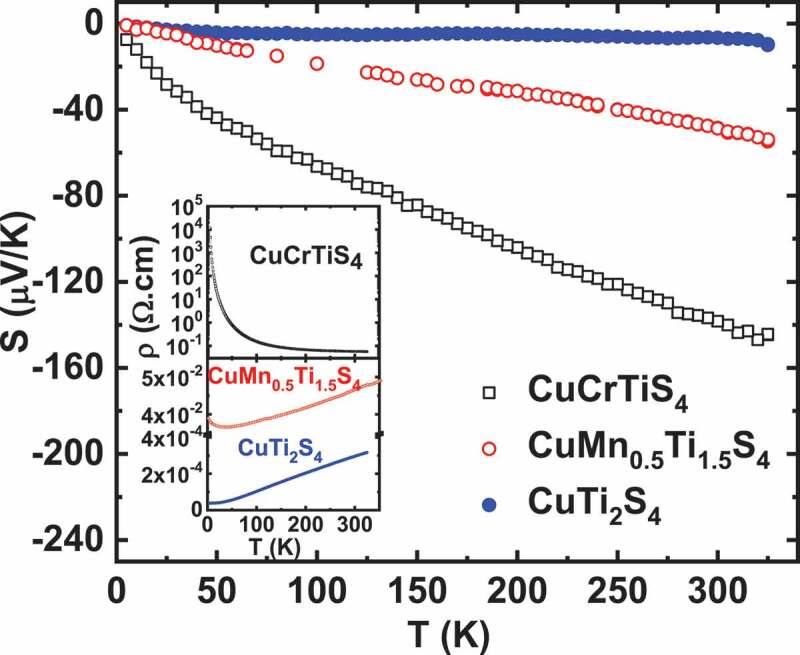
S(T) from 5 K to 325 K for CuCrTiS_4_, CuMn_0.5_Ti_1.5_S_4_ and CuTi_2_S_4_ thiospinels. Inset: Temperature dependence of electrical resistivity ρ for all the three samples

**Figure 5.**
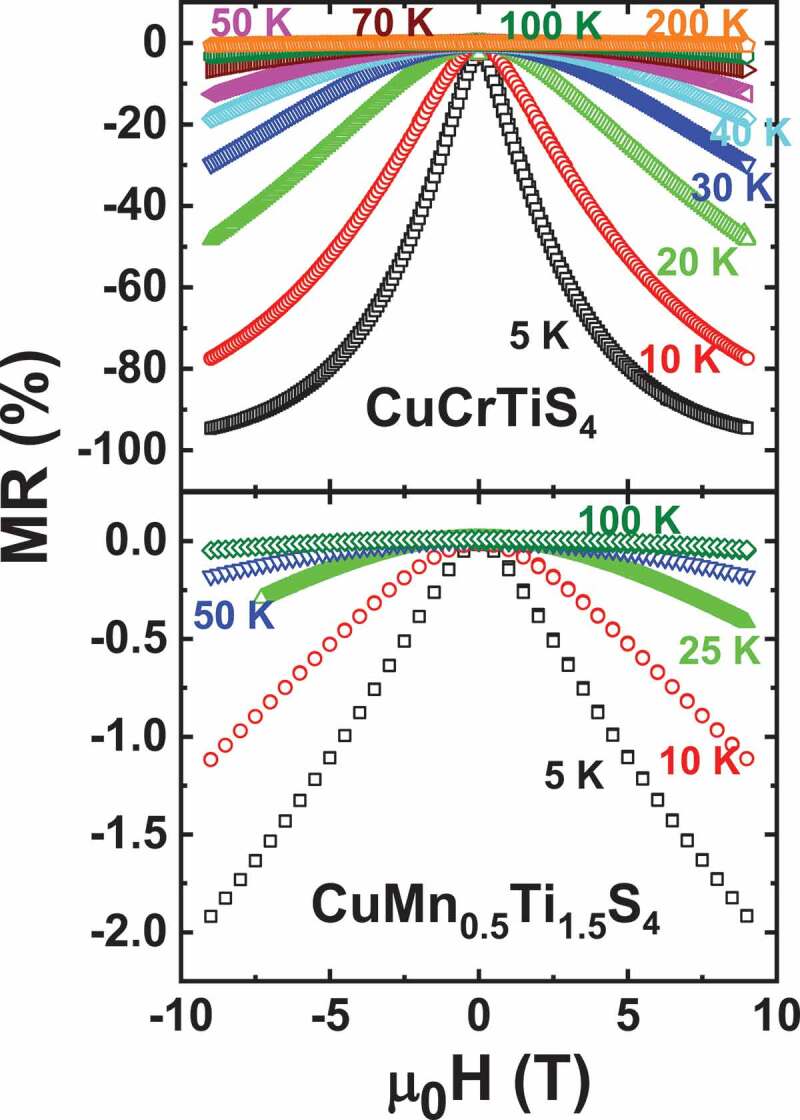
Isothermal Magnetoresistance curves of CuCrTiS_4_ (top) and CuMn_0.5_Ti_1.5_S_4_ (bottom) samples. Corresponding T values are shown in the graph

**Figure 6.**
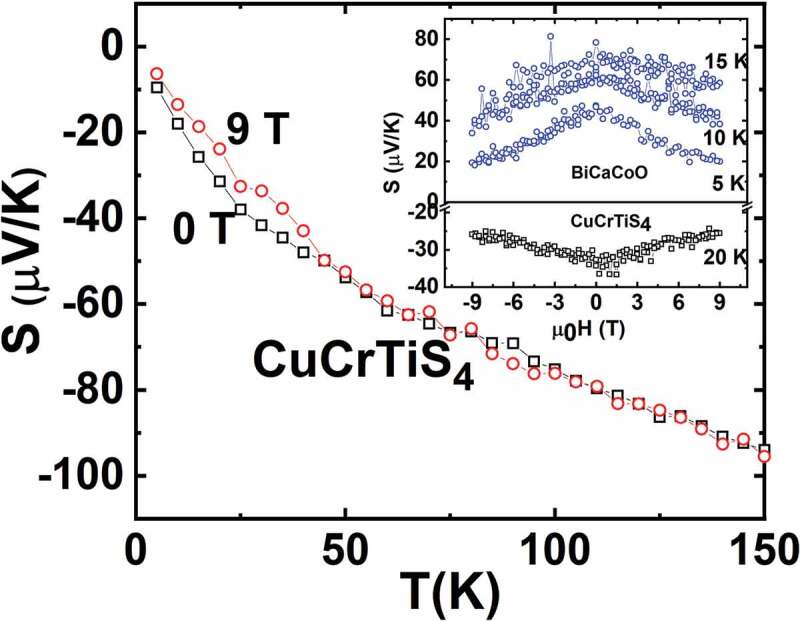
S(T) of CuCrTiS_4_ measured at 0 T and 9 T. Inset: Isothermal S(H) of CuCrTiS_4_ and BiCaCoO misfit oxide with the corresponding temperatures labeled in the graph

For spinel chalcogenides, magnetic and magnetotransport properties are determined by distribution and localization of metal ions in their tetrahedral as well as octahedral cationic sites and their oxidation states [28–30]. In this family, cubic thiospinel CuTi_2_S_4_ having Ti^3+^ and Ti^4+^ mixed valence exhibits a Pauli paramagnetic behaviour whereas a double exchange between high spin Cr^3+^ and Cr^4+^ is reported to be responsible for ferromagnetism observed in CuCr_2_S_4_ [31,32]. A solid solution exists between these two members, and the CuCrTiS_4_ thiospinel having half-filled t_2g_ Cr^3+^ and empty t_2g_ Ti^4+^ shows a paramagnetic behaviour down to low temperature (Figure 3). A spin glass transition occurs at T = 8 K [33]. Fitting susceptibility data with Curie-Weiss law shows a slight upward deviation of the experimental data below T = 75 K (inset of Figure 3). This points to small antiferromagnetic fluctuations occurring due to magnetic interaction among Cr^3+^ ions. While its magnetic properties are governed by Cr^3+^, Ti ions mainly contribute to carriers conduction. To describe transport properties for T < 75 K, a variable range hopping model (VRH) was employed for this system [21]. Despite the metallicity and rather small *S* value of both end members, i.e. ferromagnet CuCr_2_S_4_ (S = +16 µV/K) and Pauli paramagnet CuTi_2_S_4_ (S = −12 µV/K) [34], a thermopower as high as −140 µV/K at 300 K is observed in CuCrTiS_4_ (Figure 4). Due to a small departure from the stoichiometry of the compound, a small fraction of Ti^3+^ ions having slightly filled t_2g_ orbitals remains in the system, which leads to large negative *S*. A giant MR, reaching −95% at 5 K for 9 T is observed [21] (top panel of Figure 5). This could be attributed to the gradual alignment of neighboring magnetic moments of Cr^3+^ ions with the application of external magnetic field. Such a large negative MR in the VRH regime is similar to the behaviour observed in colossal magneto-resistive (CMR) manganites [35]. So MR in the whole T range was scaled as a function of $\left(1-B_{J}^{2}\right)$, where *B_J_* is the Brillouin function (in [21]), with S = J = 3/2. This confirms that paramagnetic Cr^3+^ ions progressively align themselves as the temperature is lowered and give rise to giant negative MR. The effect of external magnetic field on *S* can be observed below 45 K as shown in Figure 6. A large MTEP with absolute *S* decreasing from 34 µV/K at 0 T to 25 µV/K at 9 T for a typical temperature T = 20 K is obtained (inset of Figure 6). This *S* lowering can be attributed to the gradual alignment of Cr^3+^ spins, which decreases the magnetic entropy. For comparison, the properties of the metallic thiospinel CuMn_0.5_Ti_1.5_S_4_ are reported. This compound was found to be isostructural to the cubic CuCrTiS_4_ thiospinel. As shown in Figure 3, the susceptibility is smaller in the whole T range, with a rapid increase at low T. A Curie-Weiss fitting to this T-dependent susceptibility in between 100 K and 300 K yields µ_eff_ = 5.7 µ_B_/f.u. Such a value is consistent with S = 0 for Ti^4+^ and S = 5/2 for high spin Mn^2+^ as a theoretical value µ_eff_ = 5.9 µ_B_ is calculated for a high spin d^4^ cation. Thus, in both CuCrTiS_4_ and CuMn_0.5_Ti_1.5_S_4_, the Ti cations are tetravalent, whereas Cr is trivalent for the former and Mn divalent for the latter. CuMn_0.5_Ti_1.5_S_4_ is metallic with a small upturn of electrical resistivity below 50 K (inset of Figure 4), the temperature below which the χ values strongly increase. In this T range, below 50 K, a small negative MR appears, reaching ~ −2% in 9 T at 5 K (bottom panel of Figure 5). For this metallic thiospinel, the thermopower, with values in between those of CuCrTiS_4_ and CuTi_2_S_4_, exhibits negative values, varying almost linearly with temperature as expected for a metal (Figure 4). In clear contrast with CuCrTiS_4_ which exhibits VRH transport, only a very small MTEP is detected (not shown), showing the major role of localized paramagnetic spins on MTEP.

**Figure 7.**
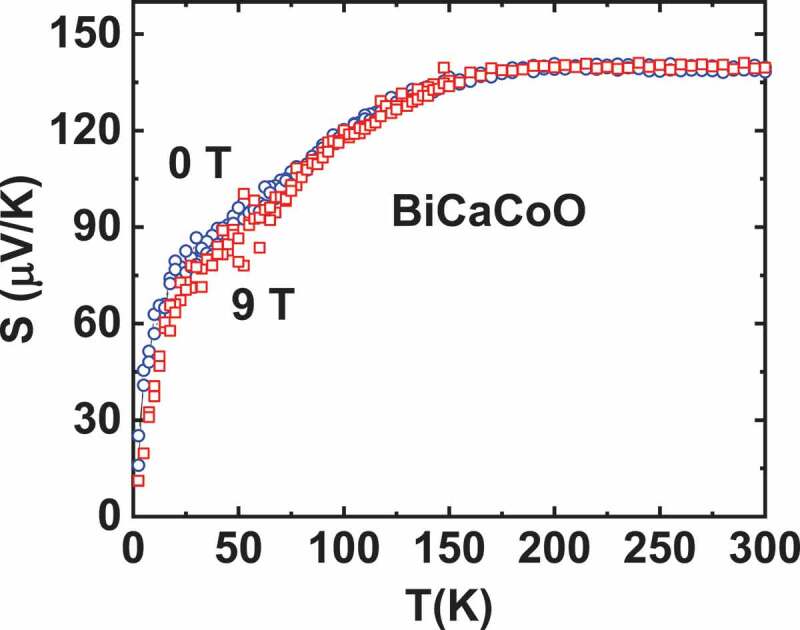
S(T) of BiCaCoO misfit oxide (in 0 and 9 T)

The positive impact of localized spins on thermopower had already been evidenced in Bi-based misfit cobaltites like [Bi*A*_2_O_4_][CoO_2_]_b1/b2_ (*A* = Ca^2+^, Sr^2+^, Ba^2+^; b_1_/b_2_ = crystallographic misfit ratio). They consist of a single layer of CdI_2_ type [CoO_2_], which is stacked with four layers of rocksalt (RS) type structure [24]. Cationic substitutions in the RS type layers are responsible for a doping of the [CoO_2_] layer by a mechanism of charge transfer and a modification of the positive charge in RS type layer, which is manifested through a change in the misfit ratio (b_1_/b_2_). These materials do not show any magnetic ordering and remain paramagnetic down to 2 K [36]. Figure 7 depicts *S*(T) data for [Bi_1.7_Co_0.3_Ca_2_O_4_][CoO_2_]_1.67_ abbreviated as BiCaCoO. Within the temperature range 20–125 K, a linear variation of *S* is observed. The large value of the slope in the linear region below 20 K reflects strong electronic correlations in accordance with the high value of the Sommerfeld coefficient γ and the universality of the ratio S/(Tγ) [7,12]. As far as MTEP is concerned, a strong magnetic field dependence of *S* is observed for BiCaCoO [24] below ~ 125 K: as shown in the inset of Figure 6, *S* is dramatically reduced as the magnetic field is applied. This decrease in *S* can be attributed to the fact that as magnetic field increases, paramagnetic spins gradually align and magnetization (M) is increased leading to the loss of entropy. A maximum decrease of 60% in *S* is observed for 9 T at 5 K. A large negative MR is also observed reaching −87% for 7 T at 2.5 K for BiCaCoO. All the *S*(H) curves below 20 K can be scaled down as a function of H/T and fitted by a Brillouin function [12]. Such a scaling behaviour confirms the freezing of spin fluctuations and hence the reduction of entropy in the system, which results in the reduction of *S*. It must be mentioned for comparison that in the metallic and Pauli paramagnet BiBaCoO misfit, no MTEP is observed [24,36], as discussed for thiospinels. MR and MTEP are observed only for localized carriers, with a large enough susceptibility associated with a Curie-Weiss behaviour.

At room temperature, no MTEP is observed in any of these misfit materials. However, the presence of magnetic cations still plays a role, as S at room temperature is mainly dependent on the formal valency of Co in [CoO_2_] layers, and on the fact that these Co cations carry a spin as shown by the generalized Heikes formula [37,38]. The *S* data around 300 K can be described by:

$$S=-\frac{k_{B}}{\left|e\right|}\ln\left[\left(\frac{2S_{n}+1}{2S_{n+1}+1}\right)\left(\frac{1-x}{x}\right)\right]+\frac{k_{B}}{\left|e\right|}ln\left(\Gamma_{orb}\right)$$

where S_n_ and S_n+1_ are the spins of the transition metal M^n+^ and M^(n+1)+^, respectively, x is the carrier concentration and г_orb_ the orbital degeneracy. In the case of Bi-based misfits, both spin and orbital degeneracy terms related to low spin Co^3+^ (S = 0) and low spin Co^4+^ (S = ½) have to be taken into account to describe its room temperature *S* [39,40]. Taking into account the lifting of the t_2g_ degeneracy [40,41] the spin and orbital terms correspond to ln (2) = ~ 60 µV/K [8,40], a sizable fraction of the thermopower, ~ 140 µV/K at 300 K for x = 0.33 (Figure 7).

### 3.2. Ferromagnetic ruthenates

**Figure 8.**
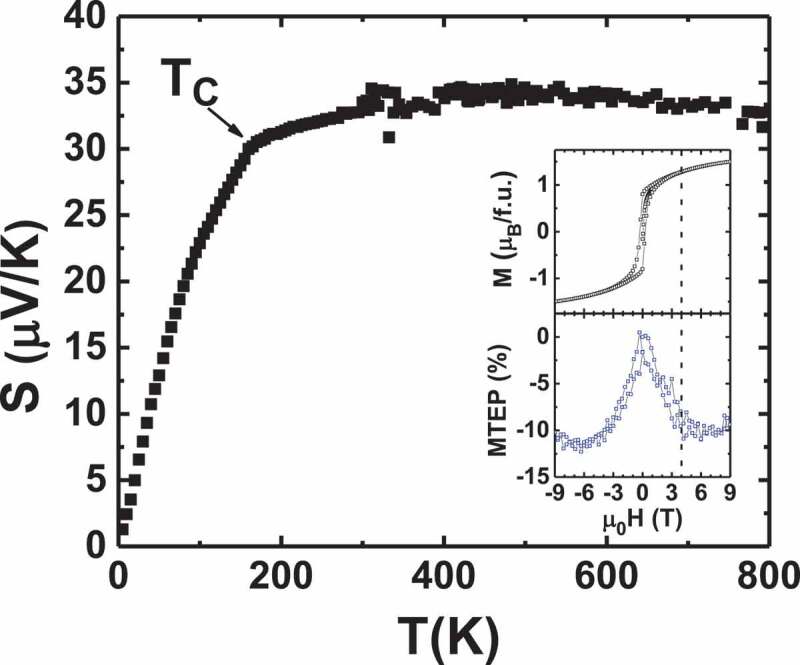
S(T) of SrRuO_3_ up to 800 K. Inset: Isothermal magneto-thermopower MTEP% = 100 x [(S(*H*) – S(*H = 0*))/S(*H = 0*)] and M(H) curve of SrRuO_3_ at a typical temperature T = 20 K

**Figure 9.**
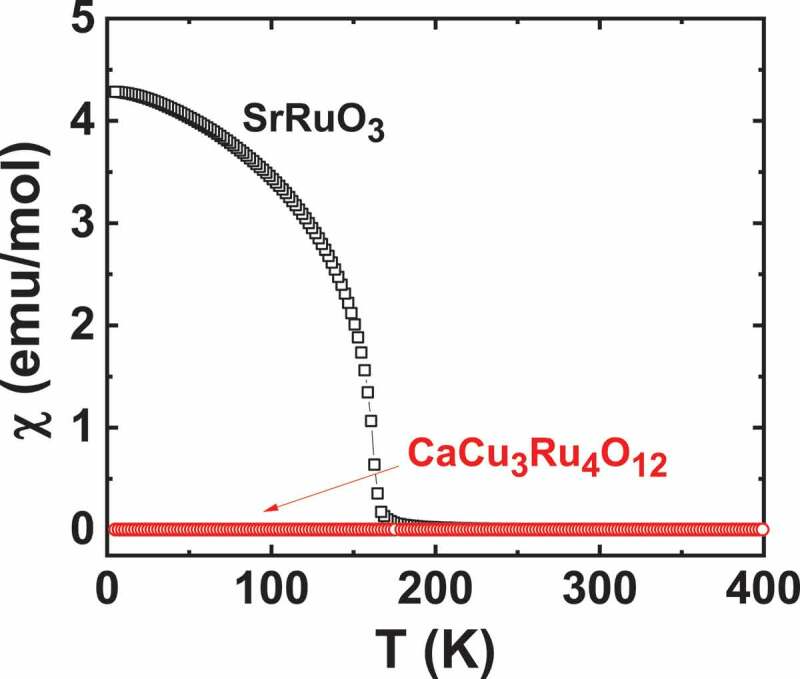
Temperature dependence of χ for SrRuO_3_ (in 0.1T) and CaCu_3_Ru_4_O_12_ sample (in 1T)

In the case of ruthenium oxides, the role of the spin entropy and magnetism can be investigated for different magnetic states due to the diversity of magnetic behaviour in these most often metallic oxides. The Seebeck coefficients of different ruthenium oxides exhibiting ferromagnetism (SrRuO_3_), paramagnetism (CaRuO_3_) and Pauli paramagnetism (quadruple perovskites) have been measured. SrRuO_3_ is a ferromagnetic metal (T_C_ ~ 160 K) where Ru takes a d^4^ electronic configuration [42]. In this perovskite structure, a low-spin (S = 1) state of Ru^4+^ is favoured due to the large crystal field splitting between e_g_ and t_2g_ orbitals in the presence of octahedrally connected oxygens [43]. A close look at Figure 8 reveals that S(T) for SrRuO_3_ does not exhibit the expected linear dependence for a typical Drude metal, and that magnetism plays a role with an accident observed near T_C_ ~ 160 K. In the case of the paramagnetic CaRuO_3_ [44], the evolution is smoother up to high temperature. Calculation of the spin only term in Heikes formula using spins of Ru^5+^ (S = 3/2) in matrix of Ru^4+^ (S = 1) and Ru^3+^ (S = 1/2) in the matrix of Ru^4+^ gives *S* value of 25 µV/K and 35 µV/K, respectively, which is very close to the measured *S* for SrRuO_3_ and related ruthenates around 300 K [25]. Above T_C_, S is driven by a constant spin entropy. This model is much too simple, and a more rigorous calculation has shown that in the case of Sr_2_RuO_4_, the orbital entropy is quenched up to 1200 K, leading to a spin entropy-dominated Seebeck coefficient below 1200 K [45]. In addition, a negative MTEP is also observed below T_C_ for SrRuO_3_ (inset of Figure 8 for T = 20 K). This field dependence of S follows the magnetization curve M(H) (Figure 9): a gradual increase of external magnetic field makes the spins more aligned up to its saturation magnetization (*M*_S_) (to be compared to M(H), inset of Figure 8), leading to a decrease of *S*. From the inset of Figure 8, when the saturation magnetization  *M*_S_ is reached, the MTEP becomes constant. The magnitude of the SrRuO_3_ MTEP is similar to the one measured in the ferromagnetic CaRu_0.8_Sc_0.2_O_3_ (−15% at 30 K) [46].

### 3.3. Pauli paramagnetic ruthenates

**Figure 10.**
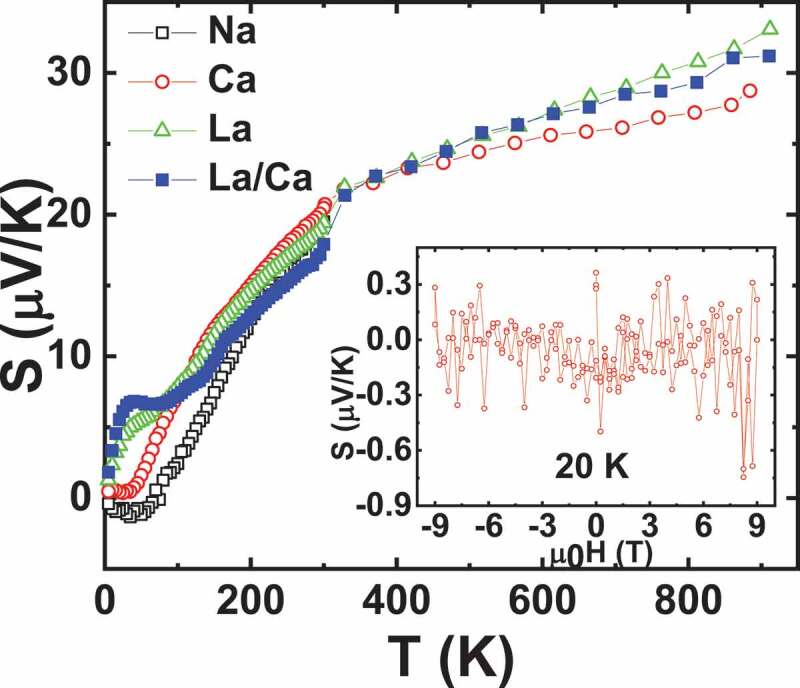
T-dependent Seebeck coefficient S for all the quadruple perovskite samples under investigation. Inset: Isothermal S(H) of CaCu_3_Ru_4_O_12_ at 20 K showing negligible MTEP

**Figure 11.**
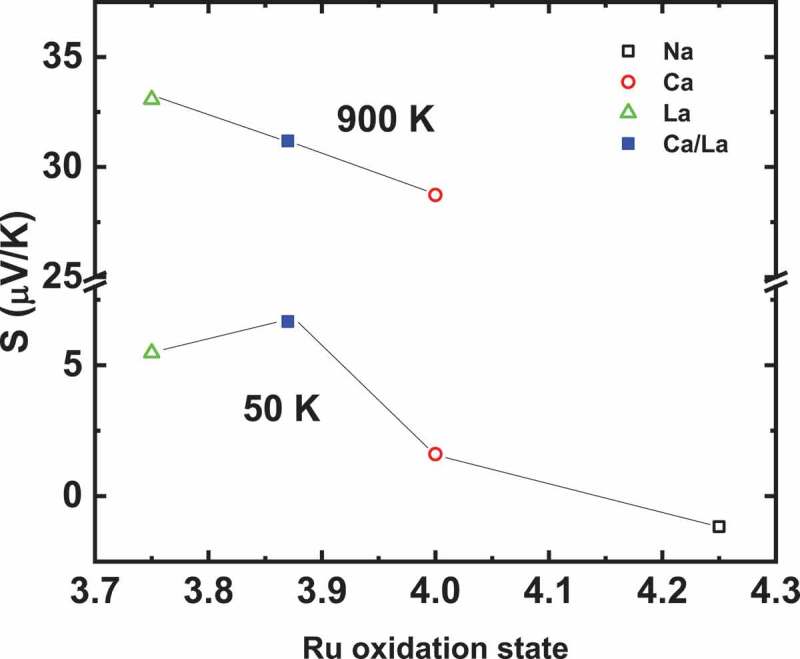
S of quadruple perovskites at 50 K and 900 K

To compare with these perovskites, *S* and ρ measurements of a group of quadruple perovskite (QP) samples *D*Cu_3_Ru_4_O_12_ (*D*= Na, Ca, Ca_0.5_La_0.5_, La) having Ru oxidation state ranging from 3.75 (D = La^3+^) to 4.25 (D = Na^+^) and presenting Pauli like behaviours (Figure 9) were performed. Density of states calculations in these materials shows that the influence of Cu states can only be observed far below *E*_F_, and hence have no effect on the transport properties [47]. ρ(T) measurements up to 900 K for LaCu_3_Ru_4_O_12_ exhibit a monotonic increase of ρ, almost linearly with T without any saturation (Figure 2 in [26]), consistently with the bad metal behaviour observed in many ruthenates such as SrRuO_3_ or Sr_2_RuO_4_ [48–50]. Even though *S* values of all these QP and single perovskite SrRuO_3_ are similar (~32 µV/K) at the highest temperature of 900 K, the evolution of S(T) differs with a gradual increase in the case of QP, with a much smaller slope than for SrRuO_3_ (Figures 10 and Figures 8). In fact, the saturation *S* value of 32 µV/K for SrRuO_3_ is reached at T ~ 200 K whereas around this temperature all the *S* curves of the QP group almost merge to 12 − 15 *μ*V*/*K. At lower temperatures, there is a substantial change in the value of *S* of *D*Cu_3_Ru_4_O_12_ and the values of S depend on the *M* value of the samples, with the smallest Seebeck coefficient measured for *Na*Cu_3_Ru_4_O_12_ (Figure 11) which possesses the smallest Pauli susceptibility. For T > 200 K, all *S* curves converge and almost a similar *S* value of all the samples is observed in the temperature range 200–900 K. So formal Ru oxidation state has no effect on *S* in these samples. A monotonic increase in *S* up to high temperature can be rather related to classical metal-like picture following Mott’s formula, where band structure plays a significant role. MTEP measurements show no significant effect of external magnetic field on *S* in these Pauli paramagnets as exemplified for CaCu_3_Ru_4_O_12_ in the inset of Figure 10.

### 3.4. Diluted paramagnetism in a telluride, a Kondo effect?

**Figure 12.**
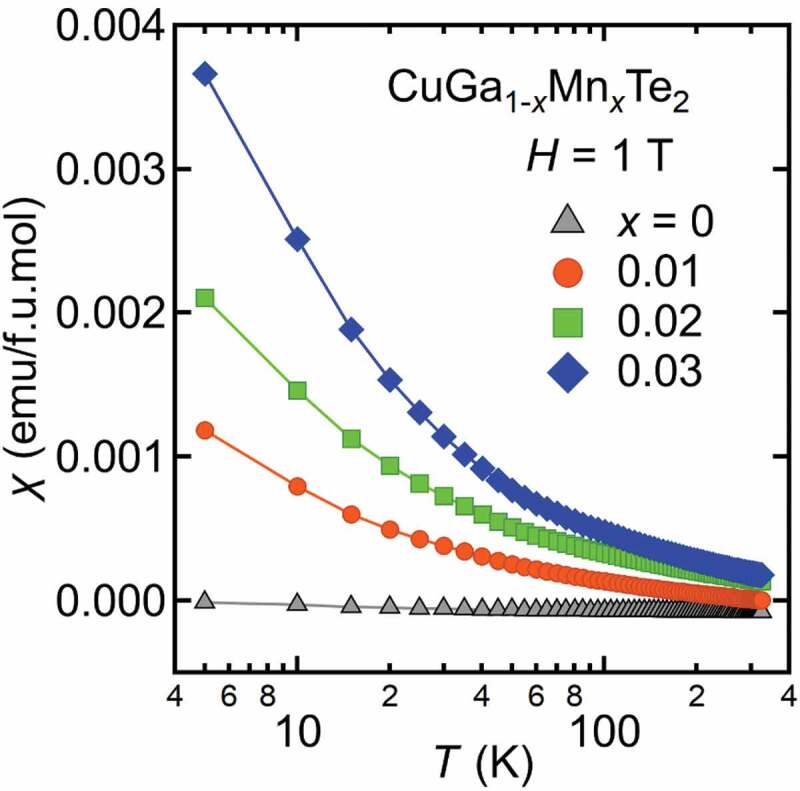
Magnetic susceptibility of CuGa_1-*x*_Mn*_x_*Te_2_ per Formula-Unit mol

**Figure 13.**
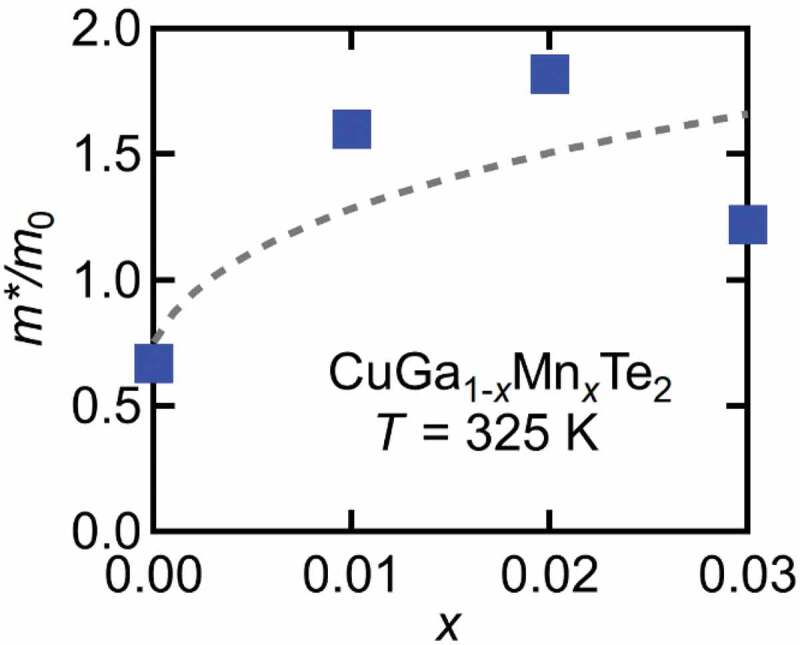
Effective mass m* with respect to the free electron mass *m*_0_ of CuGa_1-*x*_Mn*_x_*Te_2_ derived from a parabolic band model. Broken line is guide for eye

**Figure 14.**
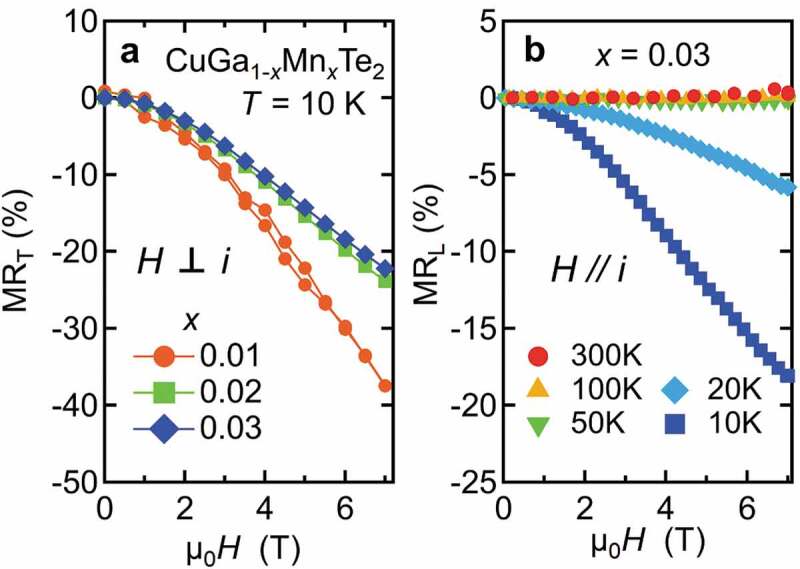
(a) Transverse magnetoresistance (MR_T_) of CuGa_1-*x*_Mn*_x_*Te_2_ measured at T = 10 K, where magnetic field is perpendicularly applied to current. (b) Longitudinal magnetoresistance (MR_L_) of CuGa_0.97_Mn_0.03_Te_2_ where magnetic field is parallel to current

The last example is CuGa_1-*x*_Mn*_x_*Te_2_ which is a paramagnetic system with no evidence of magnetic order observed at least down to 5 K (Figure 12). Mn^2+^ doping in the pristine system increases hole concentration. As a result, smaller electrical resistivity ρ is observed in *x* = 0.03 as compared to *x* = 0. But in spite of increasing in carrier concentration, relatively high TEP is observed in Mn-doped sample. Figure 13 shows the effective mass enhancement m*/*m*_0_ of CuGa_1-*x*_Mn*_x_*Te_2_, which has been derived from the Seebeck coefficients and the carrier concentrations at *T* = 325 K based on a parabolic band model. The effective mass enhances from 0.6*m*_0_ for *x* = 0 to 1.5*m*_0_ for *x* = 0.03 [51]. It has been demonstrated that the interactions between holes and the magnetic ions play a pivotal role in enhancing m*. Strong correlation between magnetic ions and holes is inferred from the magnetoresistance (MR) shown in Figure 13. At *T* = 10 K, transverse magnetoresistances MR_T_ shown in Figure 14(a) decrease significantly, reaching almost −40% for *x* = 0.01 and −20% for *x* = 0.02 and 0.03. Shown in Figure 14(b) is the longitudinal magneto resistance MR_L_ of *x* = 0.03 with field parallel to current, measured at various temperatures. MR_L_ at T = 10 K almost agrees with MR_T_ of *x* = 0.03 in Figure 14(a), which means that the large MR is intrinsic to the coupling of carriers and magnetic moments. The strong coupling has also manifested itself as the unusually large anomalous Hall effect (AHE), observed in *x* = 0.03 for T ≤ 20 K [22]. Calculation revealed a negative value of AHE constant *R*_S_ which also indicates antiferromagnetic coupling between Mn^2+^ and carriers. It is notable that the magnetoresistance in Figure 14(a) is most significant in the diluted limit (*x* = 0.01), suggesting that the on-site interaction between hole and Mn^2+^ moment is responsible, rather than the Mn-Mn inter-site couplings.

**Figure 15.**
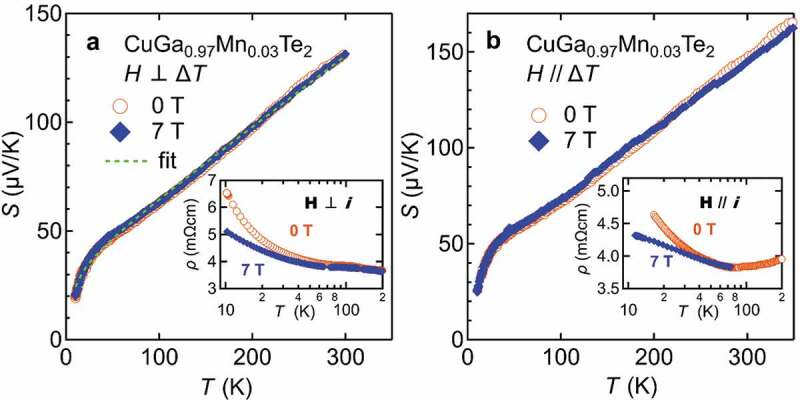
Seebeck coefficient as a function of temperature of CuGa_0.97_Mn_0.03_Te_2_ measured under µ_0_*H* = 0 and 7 T. The field is perpendicular to heat flow in (a), while parallel in (b) . Inset shows electrical resistivity under µ_0_*H* = 0 T and 7 T with current perpendicular to field in (a), and parallel to field in (b). Broken line in (a) shows a fit with the Kondo-model

This interpretation is in accordance with the results of magnetic susceptibility. Magnetic susceptibility was fitted with Curie-Weiss function, $\chi =C/(T-\theta )+\chi_{0}$ where C, θ and χ_0_ is Curie constant, Weiss temperature and the temperature-independent term, respectively. The effective magnetic moment of 5.35μ_B_ obtained for *x* = 0.03 sample is close to the Mn^2+^ moment (5.92μ_B_) with S = 5/2 and g = 2. In addition, the Weiss temperature comes out to be negative (−108.5 K), which indicates AFM interaction on Mn spins. One of the reasons of getting large negative θ can be AFM exchange between Mn^2+^ ions. But at such low Mn concentration, Mn-Mn distance is much longer, and it is difficult to imagine that Mn–Mn interaction would give rise to this large negative θ. In addition, the possibility of a direct overlap of 3d states of Mn is quite low in this case which rules out the contribution of Mn-3d impurity band to the large *S* (Figure 15). This large negative θ and large *S* can arise due to Kondo-type interaction between magnetic impurities and carriers [52]. The Kondo model was applied by Osinniy et al. to explain large TEP of ferromagnetic Ga_1-*x*_Mn*_x_*As [53]. Kondo interaction is a process of compensating the magnetic moments of impurities by forming spin-singlet states with carrier electrons. In the low temperature limit, this effect eliminates the magnetic entropies of impurities, while the entropies are transferred to carrier electrons with enhanced effective mass. As a result, the Seebeck coefficient increases with the Kondo-type interaction. Indeed, enhanced effective carrier mass m* has been observed in CuGa_1-*x*_Mn*_x_*Te_2_, as shown in Figure 12.

Figure 15 depicts the temperature dependence of *S* and electrical resistivity measured under zero and applied field of 7 T. Electrical resistivity *ρ* increases rapidly at low temperatures. Under magnetic field of µ_0_H = 7 T, this increase is significantly suppressed, as shown in the insets of Figure 15(a,b). This infers that the contribution of magnetic moment and carriers through Kondo-like mechanisms plays an important role in the transport properties of CuGa_1-*x*_Mn*_x_*Te_2_. It was suggested that the Kondo-type interaction can lead the temperature dependent Seebeck coefficient as: *S*(*T*) = *aT* + *S*_0_*T*/(*T* + *T*_0_), with *a, S*_0_, and *T*_0_ are parameters [52,53]. The broken line in Figure 15(a) is the fit with the Kondo model. The model appears to explain the overall behaviour. On the other hand, as shown in the main panel of Figure 15, *S* is hardly affected by the application of field, possibly because of the too small Zeeman energy of the field of 7 T compared to the antiferromagnetic coupling between carrier and Mn^2+^ moment, of the order of *θ* = −100 K. This contradicting behaviour has recently also been observed in Sr_2_Fe_1+x_Re_1-x_O_6_ double perovskites [54]. It must be emphasized that the thermopower depends on the entropy and on a transport term associated with the band structure [10]. As shown in all the examples presented here, MTEP is associated with MR, but on the other hand the presence of MR is not sufficient to ensure a MTEP effect due to the complex interplay between the entropic and transport terms.

**Figure 16.**
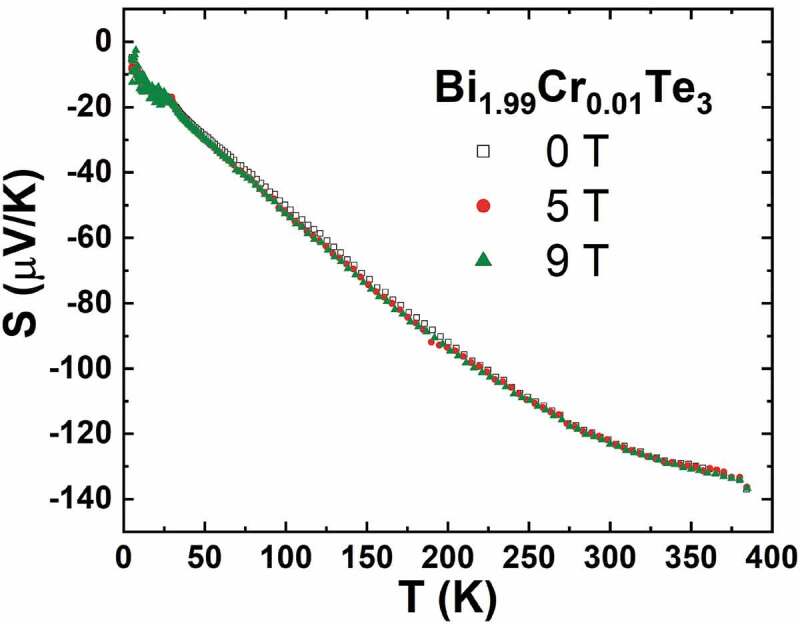
Seebeck coefficient as a function of temperature of Bi_1.99_Cr_0.01_Te_3_ measured under µ_0_*H* = 0 T, 5T and 9 T

A second similar example is the Cr-doped Bi_2_Te_3_ tetradymite system. Bi_2_Te_3_ with diluted Cr doping, as well as with transition-metal TM atoms (V, Mn and Fe), have been found to exhibit ferromagnetic transitions, with Cr doping exhibiting relatively high transition temperatures near ~240 K. An effective magnetic moment of 3.53μ_B_ is obtained for Bi_1.99_Cr_0.01_Te_3_ consistent with the Cr^3+^ moment (3.87 μ_B_). In contrast to the Mn^2+^ doping in CuGa_1-*x*_Mn*_x_*Te_2_, the Cr^3+^ doping does not affect the carrier concentration, as predicted theoretically and confirmed experimentally [23,55]. But a TEP enhancement is observed in the case of Bi_1.99_Cr_0.01_Te_3_ compared to the pristine composition and is associated with an increase of the weighted mobility μ_w_ from 73.6 cm^2^ V^−1^ s^−1^ to 76.3 cm^2^ V^−1^ s^−1^ in the doped sample with 1%mol Cr. This representative quality parameter of the charge transport can be defined as $\mu_{W}=\mu \left(m^{*}/m_{O}\right){\;}^{3/2}$, where μ is the electron mobility, $m^{*}$ is the effective mass and $m_{O}$ is the electron mass, and the increasing of this parameter supported that an additional mechanism, such as the carrier-magnetic moment interaction, was effective and increased the TEP. As evidence, the non-magnetic isovalent Ga^3+^ ions substitution for Bi^3+^ is compared with the same doping level with magnetic Cr^3+^. Only the magnetic doping with Cr led to an increase of the TEP. Bi_1.99_Cr_0.01_Te_3_ is reported to have a narrow hysteresis loop for the magnetization curves [23]. The relatively small coercivity, (µ_0_H_c_ ≈ 0.2 T), and saturation magnetization indicates that the sample is a weak ferromagnet similar to Mn-doped Bi_2_Te_3_ [56]. However, the temperature-dependent S(T) of Bi_1.99_Cr_0.01_Te_3_ measured at 0 T, 5T and 9 T (Figure 16) revealed that the TEP is not affected by the magnetic field. It is suggested that the Bi_1.99_Cr_0.01_Te_3_ TEP enhancement is not likely due to a spin fluctuation effect, as reported for the Fe_2_V_0.9_Cr_0.1_Al_0.9_Si_0.1_ [57], but by a localized electron-magnetic moment coupling promoting a heavier effective mass m* as experimentally observed [23].

## 4. Conclusions

A comparative study of MTEP in magnetically doped chalcogenides showing different magnetic behaviour was performed. A significant negative MTEP is observed in ferromagnetic perovskite ruthenates and paramagnetic misfit cobaltites. In the case of ferromagnetic material, below T_C_, the increase of magnetization is accompanied by a decrease of the entropy of the carriers and hence $\left|S\right|$ decreases. On the other hand, no MTEP is observed in quadruple perovskites indicating that Pauli paramagnetism with a very small susceptibility value is less effective in inducing MTEP. In addition, significant MTEP is absent in CuGa_1-*x*_Mn*_x_*Te_2_ and Bi_1.99_Cr_0.01_Te_3_ where Kondo-type interaction between magnetic impurities and carriers is indicated to prevail. Spin glass system CuCrTiS_4_ thiospinel also exhibits negative MTEP like in ruthenates and misfit cobaltites. Variable range hopping behaviour of the thiospinel along with negative MR makes the system similar to mixed-valence manganites [35]. Our MTEP study reveals that, in Pauli paramagnetic materials having smallest magnetic susceptibility values, the effect of spin on *S* is negligible. In contrast magnetism contributes to enhance *S* in ferromagnetic and some paramagnetic systems, whereas, when diluted, paramagnetism induces MR through a Kondo effect at very low T, but does not induce MTEP. The low temperature magnetic susceptibility of the different materials ranges from ~ 4.10^−3^ emu/mol for Pauli quadruple perovskites and CuGa_1-x_Mn_x_Te_2_ to 0.06–0.2 emu/mol for paramagnetic misfits and thiospinels, respectively, up to 4 emu/mol in SrRuO_3_. These very different values give a lower limit (~ 10^−2^ emu/mol) below which MTEP has not been observed.

This investigation suggests that one route to enhance S for TE applications is to find new materials where localized or weakly coupled magnetic ions introduce spin entropy to the system. It is interesting to notice that for such a negative MTEP, the impact of magnetism is maximum in zero magnetic field which is very important for potential applications. The presence of MTEP is a strong indicator of the role these ions play. In that respect, ferrimagnetic systems would also be of interest for MTEP measurements as they combine non-compensated antiferromagnetically coupled ferromagnetic sublattices.

## References

[1] Bell LE. Cooling, heating, generating power, and recovering waste heat with thermoelectric systems. Science 2008;321:1457–1461. 18787160 10.1126/science.1158899

[2] Petsagkourakis I, Tybrandt K, Crispin X, et al. Thermoelectric materials and applications for energy harvesting power generation. Sci Technol Adv Mater. 2018;19:836–862. 31001364 10.1080/14686996.2018.1530938 PMC6454408

[3] Heremans JP, Jovovic V, Toberer ES, et al. Enhancement of thermoelectric efficiency in PbTe by distortion of the electronic density of states. Science. 2008;321:554–557. 18653890 10.1126/science.1159725

[4] Pei Y, Shi X, LaLonde A, et al. Convergence of electronic bands for high performance bulk thermoelectrics. Nature. 2011;473:66–69. 21544143 10.1038/nature09996

[5] Li W, Chen Z, Lin S, et al. Band and scattering tuning for high performance thermoelectric Sn1−xMnxTe alloys. J Materiomics. 2015;1:307–315.

[6] Jang H, Abbey S, Nam WH, et al. Order-disorder transition-induced band nestification in AgBiSe2–CuBiSe2 solid solutions for superior thermoelectric performance. J Mater Chem A. 2021;9:4648–4657.

[7] Behnia K, Jaccard D, Flouquet J. On the thermoelectricity of correlated electrons in the zero-temperature limit. J Phys: Condens Matter. 2004;16:5187–5198.

[8] Wang Y, Rogado NS, Cava RJ, et al. Spin entropy as the likely source of enhanced thermopower in NaxCo2O4. Nature. 2003;423:425–428. 12761545 10.1038/nature01639

[9] Mori T. Novel principles and nanostructuring methods for enhanced thermoelectrics. Small. 2017;13:1702013. 10.1002/smll.201702013 28961360

[10] Peterson MR, Mukerjee S, Sriram Shastry B, et al. Dynamical thermal response functions for strongly correlated one-dimensional systems: hubbard and spinless fermion t−V model. Phys Rev B. 2007;76:125110.

[11] Koshibae W, Maekawa S. Effects of spin and orbital degeneracy on the thermopower of strongly correlated systems. Phys Rev Lett. 2001;87:236603. 11736467 10.1103/PhysRevLett.87.236603

[12] Limelette P, Hébert S, Hardy V, et al. Scaling behavior in thermoelectric misfit cobalt oxides. Phys Rev Lett. 2006;97:046601. 16907600 10.1103/PhysRevLett.97.046601

[13] Repaka DVM, Mahendiran R. Giant magnetothermopower in charge ordered Nd0.75Na0.25MnO3. Appl Phys Lett. 2013;103:162408.

[14] Wang K, Petrovic C. Large linear magnetoresistance and magnetothermopower in layered SrZnSb2. Appl Phys Lett. 2012;101:152102.

[15] Niemann AC, Gooth J, Sun Y, et al. Magneto-thermoelectric characterization of a HfTe5 micro-ribbon. Appl Phys Lett. 2019;115:072109.

[16] Hu J, Caputo M, Guedes EB, et al. Large magnetothermopower and anomalous Nernst effect in HfTe5. Phys Rev B. 2019;100:115201.

[17] Ito M, Yamashita T, Ebisu S, et al. Thermodynamic and electrical properties of CuCrTiS4. J Alloys Compd. 2014;598:133–136.

[18] Li J, Tan Q, Li JF. Synthesis and property evaluation of CuFeS2−x as earth-abundant and environmentally-friendly thermoelectric materials. J Alloys Compd. 2013;551:143–149.

[19] Tsujii N, Mori T. High thermoelectric power factor in a carrier-doped magnetic semiconductor CuFeS2. Appl Phys Exp. 2013;6:043001.

[20] Ang R, Khan AU, Tsujii N, et al. Thermoelectricity generation and electron-magnon scattering in a natural chalcopyrite mineral from a deep-sea hydrothermal vent. Angew Chem Int Ed. 2015;54:12909–12913. 10.1002/anie.201505517 26332260

[21] Berthebaud D, Lebedev OI, Maignan A, et al. Magnetothermopower and giant magnetoresistance in the spin-glass CuCrTiS4 thiospinel. J Appl Phys. 2018;124:063905.

[22] Ahmed F, Tsujii N, Mori T. Thermoelectric properties of CuGa1−xMnxTe2: power factor enhancement by incorporation of magnetic ions. J Mater Chem A. 2017;5:7545–7554.

[23] Vaney JB, Yamini SA, Takaki H, et al. Magnetism-mediated thermoelectric performance of the Cr-doped bismuth telluride tetradymite. Mat Today Phys. 2019;9:100090.

[24] Maignan A, Hébert S, Hervieu M, et al. Magnetoresistance and magnetothermopower properties of Bi/Ca/Co/O and Bi(Pb)/Ca/Co/O misfit layer cobaltites. J Phys: Condens Matter. 2003;15:2711–2723.

[25] Klein Y, Hébert S, Maignan A, et al. Insensitivity of the band structure of substituted SrRuO3 as probed by Seebeck coefficient measurements. Phys Rev B. 2006;73:052412.

[26] Hébert S, Daou R, Maignan A. Thermopower in the quadruple perovskite ruthenates. Phys Rev B. 2015;91:045106.

[27] Hejtmánek J, Jirák Z, Maryško M, et al. Interplay between transport, magnetic, and ordering phenomena in Sm1−xCaxMnO3. Phys Rev B. 2000;60:14057.

[28] Koroleva LI, Kessler YA, Lukina LN, et al. New magnetic semiconductors Fe1−xCr2(1−x)Sn2xS4. J Magn Magn Mater. 1996;157-158:475–476.

[29] Abramovich AI, Koreleva LI, Lukina LN. Spin-glass and reentrant spin-glass states in iron sulfospinels having dilute A and B sublattices. Phys Solid State. 1999;41:73–79.

[30] Ito M, Furuta T, Terada N, et al. Relaxation of magnetization in spinel CuCrZrS4. Physica B. 2012;407:1272–1274.

[31] Lotgering FK. Ferromagnetism in spinels: cuCr2S4 and CuCr2Se4. Solid State Commun. 1964;2:55–56.

[32] Matsumoto N, Hagino T, Taniguchi K, et al. Electrical and magnetic properties of CuTi2S4 and CuZr2S4. Physica B. 2000;284-288:1978–1979.

[33] Nagata S, Koseki N, Ebisu S. Spin-glass in the spinel-type CuCrTiS4. Philos Mag B. 2012;92:2957–2969.

[34] Snyder GJ, Caillat T, Fleurial JP. Thermoelectric properties of chalcogenides with the spinel structure. Mater Res Innovations. 2001;5:67–73.

[35] Viret M, Ranno L, Coey JMD. Magnetic localization in mixed-valence manganites. Phys Rev B. 1997;55:8067. 10.1103/PhysRevLett.75.3910 10059762

[36] Bobroff J, Hébert H, Lang G, et al. Interplay between magnetic properties and thermoelectricity in misfit and Na cobaltates. Phys Rev B. 2007;76:100407.

[37] Doumerc JP. Thermoelectric power for carriers in localized states: a generalization of Heikes and Chaikin-Beni formulae. J Solid State Chem. 1994;110:419–420.

[38] Koshibae W, Tsutsui K, Maekawa S. Thermopower in cobalt oxides. Phys Rev B. 2000;62:6869.

[39] Hébert S, Kobayashi W, Muguerra H, et al. From oxides to selenides and sulfides: the richness of the CdI2 type crystallographic structure for thermoelectric properties. Phys Status Solidi A. 2013;210:69–81.

[40] Pollet M, Doumerc J-P, Guilmeau E, et al. Does the orbital degeneracy play any role in the high thermopower of lamellar cobaltites? J Appl Phys. 2007;101:083708.

[41] Landron S, Lepetit MB. Importance of t2g−eg hybridization in transition metal oxides. Phys Rev B. 2008;77:125106.

[42] Cao G, Chikara S, Lin XN, et al. Itinerant ferromagnetism to insulating antiferromagnetism: a magnetic and transport study of single crystal SrRu1−xMnxO3(0⩽x<0.60). Phys Rev B. 2005;71:035104.

[43] Kobayashi H, Nagata M, Kanno R, et al. Structural characterization of the orthorhombic perovskites: [ARuO3 (A = Ca, Sr, La, Pr)]. Mater Res Bull. 1994;29:1271.

[44] Cao G, McCall S, Shepard M, et al. Thermal, magnetic, and transport properties of single-crystal Sr1−xCaxRuO3 (0<~x<~1.0). Phys Rev B. 1997;56:321.

[45] Mravlje J, Georges A. Thermopower and entropy: lessons from Sr2RuO4. Phys Rev Lett. 2016;117:036401. 27472124 10.1103/PhysRevLett.117.036401

[46] Yamamoto TD, Taniguchi H, Yasui Y, et al. Magneto-thermopower in the weak ferromagnetic oxide CaRu0.8Sc0.2O3: an experimental test for the kelvin formula in a magnetic material. J Phys Soc Jp. 2017;86:104707.

[47] Xiang H, Liu X, Zhao E, et al. First-principles study on the conducting mechanism of the heavy-fermion system CaCu3Ru4O12. Phys Rev B. 2007;76:155103.

[48] Allen PB, Berger H, Chauvet O, et al. Transport properties, thermodynamic properties, and electronic structure of SrRuO3. Phys Rev B. 1996;53:4393. 10.1103/physrevb.53.4393 9983992

[49] Klein L, Dodge JS, Ahn CH, et al. Transport and magnetization in the badly metallicitinerant ferromagnet SrRuO3. J Phys: Condens Matter. 1996;8:10111–10126.

[50] Tyler AW, Mackenzie AP, Nishizaki S, et al. High-temperature resistivity of Sr2RuO4: bad metallic transport in a good metal. Phys Rev B. 1998;58:R10107.

[51] The values m* shown here are larger than those reported in ref. [17], where the degenerate limit has been assumed. The present calculation based on a parabolic band model suggested that the Lorenz numbers of CuGa_1-*x*_Mn*_x_*Te_2_ are *L* = 1.5 to 1.8×10^−8^ WΩK^−2^, thereby suggesting the degenerate limit was not appropriate for this case.

[52] Kondo J. Theory of dilute magnetic alloys. Solid State Phys. 1970;23:183–281.

[53] Osinniy V, Dybko K, Jedrzejczak A, et al. Thermoelectric studies of electronic properties of ferromagnetic GaMnAs layers. Semicond Phys, Quantum Electron Optoelectron. 2008;11:257–265.

[54] Maignan A, Martin C, Lebedev OI, et al. Sr2Fe1+xRe1−xO6 double perovskites: magnetoresistance and (magneto)thermopower. Chem Commun. 2019;55:5878–5881. 10.1039/c9cc00926d 31045189

[55] Zhang JM, Ming W, Huang Z, et al. Stability, electronic, and magnetic properties of the magnetically doped topological insulators Bi2Se3, Bi2Te3, and Sb2Te3. Phys Rev B. 2013;88:235131.

[56] Hor YS, Roushan P, Beidenkopfet H, et al. Development of ferromagnetism in the doped topological insulator Bi2−xMnxTe3. Phys Rev B. 2010;81:195203.

[57] Tsujii N, Nishide A, Hayakawa J, et al. Observation of enhanced thermopower due to spin fluctuation in weak itinerant ferromagnet. Sci Adv. 2019;5:5935. 10.1126/sciadv.aat5935 PMC6386555 30801005

